# Normalisation process theory: a framework for developing, evaluating and implementing complex interventions

**DOI:** 10.1186/1741-7015-8-63

**Published:** 2010-10-20

**Authors:** Elizabeth Murray, Shaun Treweek, Catherine Pope, Anne MacFarlane, Luciana Ballini, Christopher Dowrick, Tracy Finch, Anne Kennedy, Frances Mair, Catherine O'Donnell, Bie Nio Ong, Tim Rapley, Anne Rogers, Carl May

**Affiliations:** 1 Research Department of Primary Care and Population Health, University College London, Upper Floor 3, Royal Free Hospital, Rowland Hill Street, London NW3 2PF, UK; 2 Division of Clinical & Population Science and Education, Mackenzie Building, University of Dundee, Kirsty Semple Way, Dundee, DD2 4AD, UK; 3 School of Health Sciences, University of Southampton, Highfield, Southampton, SO17 1BJ, UK; 4 Department of General Practice, 1 Distillery Road, National University of Ireland, Galway, Ireland; 5 Agenzia Sanitaria Regionale, Bologna, Italy; 6 School of Population, Community and Behavioural Sciences, B121 Waterhouse Buildings, University of Liverpool, Liverpool L69 3GL, UK; 7 Institute of Health and Society, Newcastle University, 21 Claremont Place, Newcastle upon Tyne NE2 4AA, UK; 8 National Primary Care Research and Development Centre, University of Manchester, Williamson Building, Oxford Road, Manchester M13 9PL, UK; 9 General Practice & Primary Care, University of Glasgow, 1 Horselethill Road, Glasgow G12 9LX, UK; 10 Arthritis Research Campaign National Primary Care Centre, Primary Care Sciences Keele University, Keele, Staffordshire, ST5 5BG, UK; 11 Faculty of Health Sciences, University of Southampton, University Road, Southampton, SO17 1BJ, UK

## Abstract

**Background:**

The past decade has seen considerable interest in the development and evaluation of complex interventions to improve health. Such interventions can only have a significant impact on health and health care if they are shown to be effective when tested, are capable of being widely implemented and can be normalised into routine practice. To date, there is still a problematic gap between research and implementation. The Normalisation Process Theory (NPT) addresses the factors needed for successful implementation and integration of interventions into routine work (normalisation).

**Discussion:**

In this paper, we suggest that the NPT can act as a sensitising tool, enabling researchers to think through issues of implementation while designing a complex intervention and its evaluation. The need to ensure trial procedures that are feasible and compatible with clinical practice is not limited to trials of complex interventions, and NPT may improve trial design by highlighting potential problems with recruitment or data collection, as well as ensuring the intervention has good implementation potential.

**Summary:**

The NPT is a new theory which offers trialists a consistent framework that can be used to describe, assess and enhance implementation potential. We encourage trialists to consider using it in their next trial.

## Background

### Complex interventions

Understanding, developing and evaluating complex interventions is essential for improving health and healthcare. Ten years ago, the Medical Research Council (MRC) published its highly influential framework for developing and evaluating interventions that 'are built up from a number of components, which may act both independently and inter-dependently' [[1], page 2]. The MRC framework has been extended and refined [2,3], emphasising, for example, that the early phases of a trial should be seen as iterative rather than linear [2,3]; that both intervention development and evaluation require a strong theoretical foundation [4]; that detailed descriptions of the intervention and the context of the evaluation are needed [2,5]; that modelling to estimate the potential benefits is important before proceeding to a trial [6,7]; and that qualitative methods can assist with understanding the processes involved in the intervention and evaluation [8,9].

The revised framework has proved invaluable for health service researchers, but there remain substantial problems in the design and conduct of complex interventions. For example, recruitment to trials remains problematic; a review of trials funded by the UK's Health Technology Assessment or the Medical Research Council found that less than one third of funded trials recruited up to their original target [10].

While the factors contributing to successful recruitment were difficult to ascertain, the authors suggested that trials which were clearly grounded in existing clinical practice had a greater chance of successful recruitment, probably because recruitment for the trial did not require additional effort from its clinical collaborators. The results from such trials were also deemed to be more readily applicable to future practice.

Another substantive problem is the gap between research evidence and practice, which remains wide, with at least two recognised gaps [11-13]. The first translational gap refers to the difficulties and barriers of moving from laboratory-based basic research to clinical medicine. The second gap focuses on the gap between developing new treatments and knowledge and implementing these in practice for the patient or population groups for which they are intended. While recognition of these gaps in the research process is valuable, a third translational gap is also evident: that of using the results of such health services research to inform wider health-related policy and practice. Such implementation failures are often attributed to slow behaviour change by professionals, but there may be other good and predictable socio-organisational reasons for such failure, including time constraints during consultations and patient preference [14]. Wider societal and contextual barriers are also present, in particular engaging policymakers in the research process, where research often does not proceed within a timeline suited to that of the policy agenda. Policy makers also engage with and use research evidence in complex ways, with the context and timing in which research findings become available as important as the evidence itself [15-17].

To overcome some of these difficulties, a number of strategies have been proposed which should be heeded by researchers interested in having their findings implemented into practice and policy. These include a greater role for theoretical approaches in research focused on implementation; consideration of how new research findings are sustained in practice; and use of a wide range of methods appropriate to the policy questions and the wider social context in which they are placed [15,18]. If these wider issues are not considered during the design of a trial, there is a risk that there will be interventions which are never implemented, despite being shown to be effective. Interventions which are not implemented will not improve health or health care. This requires both researchers designing complex interventions and research funders to consider whether an intervention exhibits the required potential for future implementation into routine practice, if demonstrated to be effective. We argue that the implementation and sustainability of interventions can be considered from the very beginning of their development and evaluation by using Normalisation Process Theory (NPT).

### Normalisation Process Theory

NPT [19,20] identifies factors that promote and inhibit the routine incorporation of complex interventions into everyday practice. It also explains how these interventions work, looking not only at early implementation, but beyond this to the point where an intervention becomes so embedded into routine practice that it 'disappears' from view (i.e., it is normalised). Normalisation is not irrevocable: practices can be denormalised; for example, few people now use a typewriter. Neither is normalisation necessarily desirable: plenty of ineffective or inefficient practices are widely normalised (overprescription of antibiotics being one example) [21].

The NPT focuses on the work that individuals and groups do to enable an intervention to become normalised. There are four main components to NPT: coherence (or sense-making); cognitive participation (or engagement); collective action (work done to enable the intervention to happen); and reflexive monitoring (formal and informal appraisal of the benefits and costs of the intervention). These components are not linear, but are in dynamic relationships with each other and with the wider context of the intervention, such as organisational context, structures, social norms, group processes and conventions.

In this paper, we discuss the role of NPT in developing, evaluating and implementing complex interventions and provide worked examples (derived from our experience) of applying NPT to these tasks.

## Discussion

It is important to distinguish clearly between the intervention (which would continue if it were subsequently implemented) and the evaluation of the intervention (which would not continue). Both can be analysed using NPT, as we demonstrate below.

### Use of NPT to develop a complex intervention

#### Define the context

However efficacious an intervention is shown to be in an experimental environment, its long-term impact depends both on its effectiveness in the "real world" and on how widely it is implemented. Researchers need to consider implementation issues during the initial intervention development, including considering the context where it will be deployed and how any changes may affect the effectiveness of the planned intervention. Change often renders a well-designed intervention irrelevant: a trial of an intervention to reduce cigarette smoking in public places may be redundant if legislation is introduced prohibiting smoking in public buildings. Hence a first task for researchers is to describe the context in which the proposed intervention would be implemented and to consider any likely changes and the likely implications of these for the proposed intervention. Questions to address include defining the staff groups affected by the intervention, considering their main current and foreseeable future concerns, and determining whether the proposed intervention will fit with these concerns. Although trial reporting standards call for a description of context [22], there is little guidance on how to do this. NPT provides a framework for mapping important elements of trial context by alerting researchers to a range of relevant contextual issues.

For example, NPT has proven valuable in understanding the context into which an intervention to promote evidence-based care of patients with back pain in UK primary care was set (the ImPACT study; Table 1). The pressures of time and the complexity of the consultation shape the context in which GPs work. In relation to back pain, not all patients presented with back pain as their primary condition, and it was often mentioned only in passing by the patient. The lack of prominence of back pain within the context of the consultation meant that the ImPACT's initial intervention to promote care for back pain had low coherence (focusing on one condition made little sense to GPs within the wider context of the consultation) and thus led to low cognitive participation and collective action. NPT clarified understanding of this problem and led to changes in the design of the trial, including attaching a GP to the project who acted as a peer advisor to GP participants and reengaged their interest in using the referral template.

**Table 1:** Use of NPT in developing complex interventions

NPT Components	Questions to consider within the NPT framework	Example: NPT evaluation of the ImPACT back pain study
**Coherence**	Is the intervention easy to describe?	Participating GPs did not differentiate the new intervention from current practice and were unable to perceive the projected benefits to patients, primary care teams and physiotherapists.
	Is it clearly distinct from other interventions?	
(i.e., meaning and sense making by participants)	Does it have a clear purpose for all relevant participants?	
	Do participants have a shared sense of its purpose?	
	What benefits will the intervention bring and to whom?	
	Are these benefits likely to be valued by potential participants?	
	Will it fit with the overall goals and activity of the organisation?	
**Cognitive participation**	Are target user groups likely to think it is a good idea?	Participating GPs saw it as research (i.e., recruiting patients to the study), and peripheral to their main task of delivering patient care. Projected benefits were not obvious to the GPs so they were insufficiently motivated to invest thought and energy into changing their practice.
(i.e., commitment and engagement by participants)	Will they see the point of the intervention easily?	
	Will they be prepared to invest time, energy and work in it?	
**Collective action**	How will the intervention affect the work of user groups?	Participating GPs were expected to use a computer-based decision-support tool during consultations. Many GPs did not access the computer until after the consultation was completed. GPs were unconvinced that such a brief tool could form an appropriate basis for decisions about referral.
	Will it promote or impede their work?	
(i.e., the work participants do to make the intervention function)	What effect will it have on consultations?	
	Will staff require extensive training before they can use it?	GPs were not fully aware of the additional training received by participating physiotherapists, and did not therefore realise that the physios were well equipped to deal with emotional or psychological components of back pain.
	How compatible is it with existing work practices?	GPs already felt under pressure of time in consultations, and felt that using the decision-support tool was an unjustified additional use of time.
	What impact will it have on division of labour, resources, power, and responsibility between different professional groups?	
	Will it fit with the overall goals and activity of the organisation?	
**Reflexive Monitoring**	How are users likely to perceive the intervention once it has been in use for a while?	Despite regular feedback from the research team GPs did not perceive benefits to the new system as they did not use it enough.
(i.e., participants reflect on or appraise the intervention)	Is it likely to be perceived as advantageous for patients or staff?	
	Will it be clear what effects the intervention has had?	
	Can users/staff contribute feedback about the intervention once it is in use?	
	Can the intervention be adapted or improved on the basis of experience?	

The UK ImPACT back study aims to promote evidence-based care of patients with back pain in primary care[23]. Physiotherapists were trained to provide psychological support to patients with low back pain, and GPs were asked to use paper-based or computer-based decision-support tools to assess patients with back pain and refer those at risk of developing chronic low back pain to these specialised physiotherapists. As a result of the analysis presented here, the intervention could be modified to provide greater coherence to the GPs, and hence greater cognitive participation as well as modifications which enabled better fit with existing GP consultation practices.

The context for this intervention was UK primary care. GPs were a potential rate-limiting factor as they had to use the intervention to refer patients to the specialised physiotherapists. GPs main concerns were providing high quality care under extreme pressure of time while responding to multiple other changes in primary care. Many UK GPs reported feeling under pressure and suffering from "change fatigue", leading to a concentration on "core business" rather than "optional extras" such as research or teaching.

#### Define the intervention

The next task is to define the intervention. This is demonstrated through the example shown in Table 1. Physiotherapists were trained to provide psychological support to patients with low back pain, and GPs were asked to use paper- or computer-based decision support tools to assess patients with back pain and refer those at risk of developing chronic low back pain to these specially trained physiotherapists [23].

#### Undertake an NPT analysis of the intervention

As shown in Table 1, the intervention had low coherence for participating GPs, who mainly focused on one aspect (the decision-support tool), which did not make clinical sense to them. Because of this low coherence, there was low cognitive participation, with the GPs seeing little point to the intervention, which led in turn to low collective action (an unwillingness to invest time or energy in implementation), particularly as the intervention required a change in consultation behaviour. GPs tended to ignore the decision-support tool and to focus on the length of waiting lists when making referral decisions. As a result, patients were not referred to the enhanced physiotherapy treatment and GPs did not receive positive feedback about the new service (low reflexive monitoring).

Addressing the questions outlined in Table 1 is likely to need a mixture of literature reviews and primary data collection (e.g., observation, interviews, and/or questionnaires). This analysis allowed the researchers to redesign future interventions, improving their coherence and fit with existing consultation practices so that GPs could easily grasp what was involved, tailor their work practices and see the potential benefit for patients. For example, using NPT within ImPACT emphasised the importance of understanding the varied contexts of primary care (e.g., number of partners, communication between GPs, models of team working, use of guidelines), how to gain participation (e.g., through the use of champions) and maintain this (e.g., through peer support), highlighting the potential benefits of participation in the trial and the 'fit' with routine practice. Such insights were subsequently used in the design of another large implementation study.

**Figure 1:**
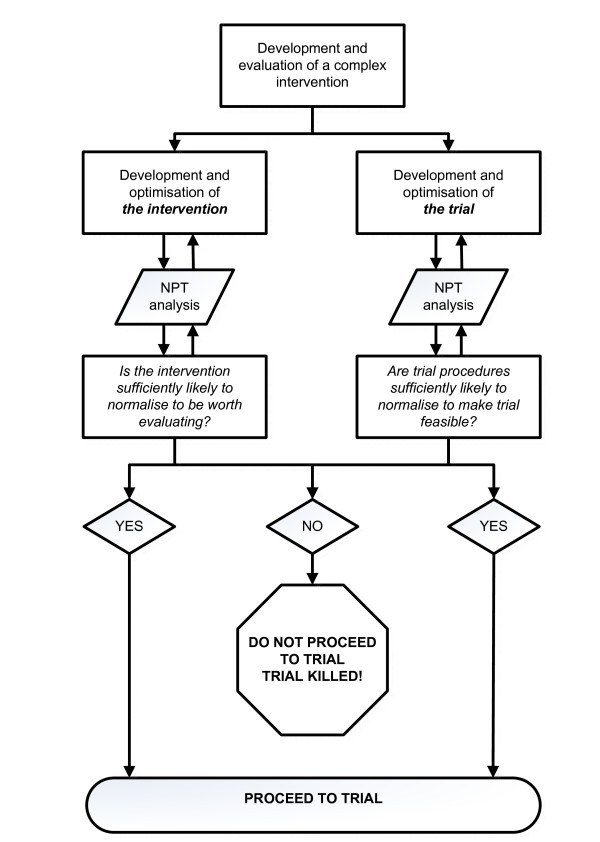
**Normalisation Process Theory (NPT) as a 'trial killer'**. Context: All important for development, evaluation and implementation.

With ImPACT, the NPT informed the redesign of the intervention for subsequent studies to enhance its potential for normalisation. Sometimes this may not be possible, and in this case researchers may need to consider whether proceeding to a more formal evaluation is justified. In some cases, the potential benefits may be sufficient to justify continuing and working on overcoming barriers to normalisation. In other cases, researchers may be better advised to abandon the intervention, rather than using scarce research funds to assess an intervention that has little chance of implementation. This is an example of NPT acting as a 'trial killer' (Figure 1). While we have discussed this in relation to the design and evaluation of trials of complex interventions, the principles can also be applied to clinical trials of new drug treatments or medical devices. If there is little likelihood of the treatment or device being normalised into either routine clinical practice or the patients' lives, for example, because of the demands the treatment regimen makes of patients [24], then the trial should be reconsidered or abandoned.

### Use of NPT to optimise evaluation of a complex intervention

NPT can also be used to guide the design of the evaluation of a complex intervention. In this paper, we focus on optimisation of trial parameters, but a similar approach can be used for other evaluative methodologies as well as clinical trials of noncomplex interventions. Table 2 presents a worked example derived from the Whole System Informing Self-management Engagement (WISE) trial [25]. The trial aimed to evaluate a whole systems approach to improving outcomes in long-term conditions through effective self-management. The intervention involved training a whole practice team to provide self-care support for patients. As part of the optimisation of trial parameters (recruitment), the study team undertook an exploratory study to help optimise the content of the training and ensure that the research protocol was acceptable to the service users, professionals and organizations targeted.

**Table 2:** Use of NPT in optimising trial parameters

NPT Components	Questions to consider within the NPT framework	Example: an NPT evaluation of the WISE trial
**Coherence**	Is the trial easy to describe?	Yes, practices understood the trial explored whether providing training to the practice team affected patient ability to self-care.
(i.e., meaning and sense making by participants)	Is it clearly distinct from other studies?	Recruitment was in two stages: practice recruitment and randomisation to either immediate training or training after 1 year, and then patient recruitment in the first year. Outcomes data collected at the level of the individual patient, so good communication about the timing of training and patient recruitment was required.
	Does it have a clear purpose for all relevant participants?	Providing self-care support may require clinicians to challenge current patient behaviours and risks disrupting existing relationships. Hence during practice recruitment the emphasis was on the benefits of the training, including development of practical strategies and improving skills which would benefit patient care.
	Do participants have a shared sense of its purpose?	
	What benefits will the trial bring and to whom?	Patients were unlikely to perceive any direct personal benefit from participation, and so financial incentives were required to improve completion of postal questionnaires. The initial informed consent process was found to be a potential 'trial killer' as it had a very negative impact on patient recruitment rates. Ethical approval was sought and obtained to simplify the process.
	Are these benefits likely to be valued by potential participants?	
	Will it fit with the overall goals and activity of the organisation?	
**Cognitive participation**	Are target user groups likely to think the trial is a good idea?	Clinicians may hold the view that their patients do not want to be self-managers, and provide evidence to challenge this. Alternatively, they may see potential benefits in reduced workload with these patient groups.
(i.e., commitment and engagement by participants)	Will they see the point of the trial easily?	
	Will they be prepared to invest time, energy and work in it?	Patients may have altruistic reasons for participating (e.g., improving future provision of self care support for others) which can be drawn on to encourage continuing participation.
**Collective action**	How will trial procedures affect the work of user groups?	Participation ensures controlled access to desired resources such as additional Cognitive Behavioural Therapy and patient information books which may incentivise participation.
	Will they promote or impede their work?	
(i.e., the work participants do to make the intervention function)	What effect will it have on consultations?	General practice staff concerns about increased time burden will need to be addressed.
	Will participation in the trial require extensive training for staff involved?	For staff, the trial provides an opportunity to have protected training time together which is unusual and appreciated but needs additional financial resources.
	How compatible is the trial with existing work practices?	Initial input is needed from practice staff to recruit patients to the trial and service support costs provided by the research body are an encouragement for practices. Once patients are recruited, the research team takes over the burden of patient follow-up.
	What impact will it have on division of labour, resources, power, and responsibility between different professional groups?	It is hard to engage GP practices with research, and sustained support from the PCT and early adopters (practices who participated first and champion the research) have been key to engaging other practices in the PCT.
	Will the trial fit with the overall goals and activity of the organisation?	
**Reflexive Monitoring**	How are users likely to perceive the trial once it's been on-going for a while?	Trainers in self care support can provide ongoing contact, feedback and help the practice access resources for their patients, so effects should be visible quickly.
	Is it likely to be perceived as advantageous for patients or staff?	
(i.e., participants reflect on or appraise the intervention)	Will it be clear what effects the study has had?	
	Can users/staff contribute feedback about study procedures?	Quick action in obtaining substantial amendments from ethics to improve trial procedures (e.g., informed consent procedure; incentives for patients; information about accessing resources for clinicians and patients) helped to ensure progress of the trial.
	Can the study procedures be adapted/improved on the basis of experience?	

The WISE trial (ISRCTN90940049 - Evaluation of the WISE (Whole System Informing Self-management Engagement) approach in primary care: improving outcomes in chronic conditions through effective self-management) [25]. The intervention involves training a whole practice team to provide self-care support to patients. An exploratory study undertaken prior to the full trial drew on NPT to help optimize the training content and sensitise research to the reaction, incorporation or rejection of the WISE approach from a service user, professional and organisational perspective.

The context for this trial was UK primary care. Contextual issues that needed consideration in planning the trial included a policy framework which supported self-management; a performance management system which incentivises GPs to achieve specific clinical targets (the Quality and Outcomes Framework) which may conflict with self-management and places a requirement on GPs to collate and submit data on a range of performance issues with subsequent implications for practice income. These data are submitted annually, making the year end an exceptionally busy time for practices so trial-related work is likely to receive lower priority at this time.

#### Define the context

Considering the context of the evaluation is as essential as considering the context of the intervention. Questions to consider include identifying systems already in place and considering how well the proposed trial procedures fit with these systems. Are any major changes to the trial context likely to occur during the trial period? For example, running a trial in a setting that is undergoing major reorganisation is unlikely to be easy, as staff may be unable or unwilling to engage with the study. As described in Table 2, consideration of the timing of patient recruitment and data collection was crucial. If these activities overlapped with the end-of-year workload of practices collecting routine clinical data for the Quality and Outcomes Framework [26], the trial could be incompatible with practice workload (a negative impact on collective action), with practices less likely to conduct the work required for the trial.

#### Define the trial parameters and consider all the different patient and professional groups likely to be affected

Researchers are used to defining the trial parameters (recruitment, randomisation, data collection, outcome measures and follow-up). Many researchers are less used to thinking about the impact of the trial procedures on the work of all the people affected by the trial, including health professionals, patients and support staff.

#### Undertake an NPT analysis of the trial parameters

Having considered all the different professional and patient groups likely to be involved in the work of the trial, the researcher needs to consider how the trial procedures will affect them. Will the trial make sense and appear relevant to those involved (i.e., will it be coherent)? Cognitive participation is likely to be enhanced if staff involved can see both immediate and longer-term benefits to patients and practice [10,27]. High levels of cognitive participation will help engage staff in collective action, but minimising the amount of work required by participants is also vital. Recruitment and data collection should be minimally disruptive of existing practices or workflows: for example, using a researcher to recruit patients may be more cost-effective than asking GPs to recruit, as GPs may struggle to remember the study amongst all the competing demands on their attention [28]. It is worth noting that trials that offer additional services are more likely to encourage clinical participation than those which ask clinicians to abstain from offering existing services or treatments which have already become normalised [29]. Reflexive monitoring can be enhanced by regular feedback, and this can be personalised (e.g., individual recruitment rates). Newsletters, too, can help foster the sense that the trial is important, reinforcing cognitive participation and strengthening collective action.

This is a second opportunity for the NPT to act as a 'trial killer'. Consideration of the impact of the trial on the work of the professionals affected may show that the proposed recruitment or follow-up rates are unfeasible.

### Implementation

Once an intervention has been proven effective, the next step is to ensure wide scale implementation, a task that often falls to people other than those who did the original development and evaluation.

#### Consider the context

People responsible for implementing a complex intervention need to know the context in which the intervention was developed and evaluated and consider what differences there are between those and the context for the planned implementation. For example, a Computer Decision Support System (CDSS) designed to help call centre workers triage emergency calls to the Ambulance Service is likely to require reconfiguration for use in a nonemergency context, where the epidemiology of health problems leading to calls will be quite different.

#### Define the intervention to be implemented

One of the problems with implementing complex interventions is defining the intervention. In the example given above of a Computer Decision Support System, is the intervention the software or the combination of the software and the staff working in the call centre?

#### Undertake an NPT analysis of the implementation

Having considered the context, defined the intervention and thought about all the different groups of staff likely to be affected by the implementation, the implementer is ready to undertake an NPT analysis. We present in Table 3 a worked example of an NPT analysis undertaken prior to implementation of robotic urological surgery in Italy. In this example, the intervention was easy to describe and distinguish from current practice as it required new technology, equipment and skills. It was likely to benefit patients and professionals at centres offering the new surgical technique, while professionals working at centres not offering it might feel disadvantaged by the potential loss of patients. The NPT analysis allowed the Health Board responsible for introducing robotic surgery to identify this as a potential problem before starting the implementation programme and to consider strategies for addressing this. Similarly, the NPT analysis identified the need for extensive training of staff in selected units, consideration of the impact of the new technology on patient referral patterns (and how this could destabilise some provider units), and the need for ongoing monitoring and feedback to units to allow them to reflect on the service offered.

**Table 3:** Use of NPT in implementing complex interventions

NPT Components	Questions to consider within the NPT framework	Example: an NPT evaluation of robotic urological surgery
**Coherence**	Is the intervention easy to describe?	The intervention is easily distinguishable from current surgical techniques by the technology involved, new skills required, new operating theatre equipment needed and higher costs of the service.
	Is it clearly distinct from other interventions?	
(i.e., meaning and sense making by participants)	Does it have a clear purpose for all relevant participants?	
	Do participants have a shared sense of its purpose?	
	What benefits will the intervention bring and to whom?	It is expected to improve the performance and the clinical outcomes of minimally invasive techniques.
	Are these benefits likely to be valued by potential participants?	
	Will it fit with the overall goals and activity of the organisation?	
**Cognitive participation**	Are target user groups likely to think the intervention is a good idea?	Professionals offered the technology are likely to be enthusiastic and prepared to invest their time and training in it.
		
(i.e., commitment and engagement by participants)	Will they see the point easily?	
		Surgeons not offered the technology might not see it as advantageous and might discourage their patients from accessing the technology, particularly as this would mean the patient transferring to another centre.
	Will they be prepared to invest time, energy and work in it?	
**Collective action**	How will the intervention affect the work of user groups?	Surgeons working in centres not offering robotic surgery may hesitate to offer this treatment option which requires onward referral of the patient and may adversely affect the surgeon-patient relationship.
	Will it promote or impede their work?	
(i.e., the work participants do to make the trial function)	What effect will it have on consultations?	
	Will staff require extensive training before they can use it?	Most surgeons do not have the necessary skills and knowledge to use the new technology. New training programmes with defined content and assessment procedures will be needed to ensure accountability and confidence.
	How compatible is it with existing work practices?	Establishing a highly specialized surgical network, where patients are referred from 'nondoers' to 'doers' and where surgical teams move between hospitals, will contribute to the development of a surgical elite, which will attract patients, resources, research resources and prestige. The impact of this will need to be monitored.
	What impact will it have on division of labour, resources, power, and responsibility between different professional groups?	
	Will it fit with the overall goals and activity of the organisation?	Centres which are offered and choose to adopt the new service are likely to view it as having a positive impact on their goals, as it is likely to increase patient numbers. However, they will have to invest resources to achieve the structural and organizational changes required and take responsibility for accommodating the expected increased flow of patients, for training programmes and for specific risk management programmes.
**Reflexive Monitoring**	How are users likely to perceive the intervention once it has been in use for a while?	Systematic review evidence details the expected clinical impact of the new technology. Clinical audit will be undertaken to determine whether the expected benefits are being achieved in routine clinical practice.
(i.e., participants reflect on or appraise the trial)		
	Is it likely to be perceived as advantageous for patients or staff?	Impact on equity of healthcare for patients and impact on development of the surgical network and surgical centres should be monitored through administrative data.
	Will it be clear what effects the intervention has had?	
	Can users/staff contribute feedback about the intervention once it is in use?	Individual centres will be encouraged to ensure users have 'ownership' of the systems around the new surgical technique (training, accreditation, patient flow) and can adapt these as appropriate.
	Can the intervention be adapted/improved on the basis of experience?	

Robotic urological surgery: a highly technological and costly form of minimally invasive surgery that introduces new skills and new patterns of action in healthcare with important repercussions on surgical services, patients' access to services, on the professional network of surgeons and on hospitals' budgets [36]. NPT analysis was undertaken from the point of view of a commissioning agency, responsible for healthcare provision across the whole local health economy, as part of implementation planning.

The context for this implementation was secondary care across the administrative region of Emilia-Romagna, Italy. Surgical urological interventions were already widely available across the region, but access to minimally invasive surgical techniques varied widely between centres. These differences are likely to be increased and accentuated by the introduction of robotic urological surgery.

## Summary

The goal of research into complex interventions is to improve health. This requires first that researchers ensure that interventions they develop and evaluate can be widely implemented and second that their evaluations provide definitive assessments of efficacy and effectiveness. NPT provides a framework that can help with both tasks. The explicit consideration of the implementation potential of an intervention is, we believe, rarely done by trialists before a trial commences. This may partially explain the 'know-do' gap between evidence about effective interventions and routine clinical practice. Moreover, NPT acknowledges that healthcare is a collective activity requiring a multitude of interactions between professionals, patients, managers and others. An intervention that appears to affect only one individual or group may, on closer inspection, require a successful chain of interactions. NPT will not solve these problems, but it can help to identify how links between participants may be affected by the intervention and how the intervention might be modified to support these interactions. This is, we believe, a clear strength of NPT compared to other approaches to implementation, which tend to focus principally on the needs of one professional group or level at a time, with less consideration given to the wider system issues. For example, theories of individual preferences (in economics) [30], intentions (in psychology) [31] and interests (in sociology) [32] support understanding of how participants in these collective activities frame behaviour. However, because such theories focus on individual and not group processes, they are inevitably much less successful in accounting for organisational processes characterised by complexity and emergence, where multiple confounders act upon behaviour. Alternatively, approaches such as Rogers' Diffusion of Innovations focus on whole systems, with little consideration of component parts [33]. Nor do other approaches to implementation consider the 'work' that needs to be done to maintain the intervention in routine practice, either by professionals or by patients [24,34]. A recent Cochrane review on the development of tailored interventions to overcome barriers to change found that such interventions were more likely to improve professional practice, but that the methods used to identify barriers and develop tailored interventions needed further development [35]. We suggest that the clear theoretical framework offered by NPT, addressing both individual and organisational level factors, can help with both these tasks.

NPT is a new theory, and we believe that it offers trialists something that has to date been lacking: a consistent framework that can be used to describe and judge implementation potential but also, importantly, to design and improve complex interventions. For this reason, it is worth building up an empirical body of work to test NPT in practice. Too many trials fail to have an impact on practice [11], a situation no trialist wants and which will not change unless interventions are developed with an explicit theoretical framework. We consider that NPT is a strong candidate framework and encourage trialists, both those designing trials of complex interventions and those designing clinical trials, to consider using it in their next trial.

## Competing interests

The authors are all members of the Normalisation Process Theory Peer Learning Group, funded by the National Institute of Health Research. The paper is a result of two residential meetings, ongoing collaboration and discussion between these meetings and our collective experience of developing and evaluating complex interventions in healthcare. All authors have completed the Unified Competing Interest form available at http://www.icmje.org/coi_disclosure.pdf (available on request from the corresponding author) and declare that they have the following competing interests: Membership of an NIHR-funded Peer Learning Group to develop and critique NPT, which funds travel and accommodation expenses for an annual meeting (all).

## Authors' contributions

All authors have contributed equally to the discussions which gave rise to the intellectual content of the paper. AK, BNO, LB, AMcF and TF contributed examples of using the NPT in developing, evaluating or implementing complex interventions from their personal experience. EM wrote the first and final drafts and acts as guarantor of the paper. CP, CO'D and ST substantially edited the final draft. All authors contributed to revisions and agreed to the final draft.

## Pre-publication history

The pre-publication history for this paper can be accessed here:

http://www.biomedcentral.com/1741-7015/8/63/prepub
